# Measuring and Valuing Health-Related Quality of Life among Children and Adolescents in Mainland China – A Pilot Study

**DOI:** 10.1371/journal.pone.0089222

**Published:** 2014-02-20

**Authors:** Fei Xu, Gang Chen, Katherine Stevens, HaiRong Zhou, ShengXiang Qi, ZhiYong Wang, Xin Hong, XuPeng Chen, HuaFeng Yang, ChenChen Wang, Julie Ratcliffe

**Affiliations:** 1 Department of Non-communicable Disease Prevention, Nanjing Municipal Center for Disease Control and Prevention, Nanjing, China; 2 The School of Public Health, Nanjing Medical University, Nanjing, China; 3 Flinders Health Economics Group, Flinders University, Adelaide, Australia; 4 Health Economics and Decision Science, University of Sheffield, Sheffield, United Kingdom; Iran University of Medical Sciences, Iran (Republic of Islamic)

## Abstract

**Background:**

The Child Health Utility 9D (CHU9D), a new generic preference-based health-related quality of life (HRQoL) instrument, has been validated for use in young people in both the UK and Australia. The main objectives of this study were to examine the feasibility of using a Chinese version of the CHU9D (CHU9D-CHN) to assess HRQoL and to investigate the association of physical activity, homework hours and sleep duration with HRQoL in children and adolescents in Mainland China.

**Methods:**

Data were collected using a multi-stage sampling method from grades 4–12 students in May 2013 in Nanjing, China. Consenting participants (N = 815) completed a self-administered questionnaire including the CHU9D-CHN instrument and information on physical activity, homework and sleep duration, self-reported health status, and socio-demographic characteristics. Descriptive and multivariate linear regression analyses were undertaken. CHU9D-CHN utility scores were generated by employing two scoring algorithms currently available for the instrument, the first derived from UK adults utilising the standard gamble (SG) valuation method and the second derived from Australian adolescents utilising the best-worst scaling (BWS) method.

**Results:**

It was found that CHU9D utility scores discriminated well in relation to self-reported health status and that better health status was significantly associated with higher utility scores regardless of which scoring algorithm was employed (both p<0.001). The adjusted mean utilities were significantly higher for physically active than inactive students (0.023 by SG, 0.029 by BWS scoring methods, p<0.05). An additional hour of doing homework and sleep duration were, separately, associated with mean utilities of −0.019 and 0.032 based on SG, and −0.021 and 0.040 according to BWS scoring algorithms (p<0.01).

**Conclusion:**

The CHU9D-CHN shows promise for measuring and valuing the HRQoL of children and adolescents in China. Levels of self-reported physical activity, homework and sleep time were important influencers of utility scores.

## Introduction

Health related quality of life (HRQoL) is a patient-based multidimensional construct that measures the impact of health or disease on physical and psychosocial functioning [1], [2]. HRQoL has been widely used not only in clinical practice but also in the evaluation of public health and health promotion interventions [3]–[5]. Identifying HRQoL associated factors could provide useful information for the implementation of relevant public health policy. Recently, increasing attention has been given to the measurement of HRQoL for children and adolescents [6]. The adolescent phase, marked by considerable physical, psychological and social changes affecting HRQoL [7], is critical for lifestyle and behavioral intervention. For example, it is now well-documented that adolescence plays an important role in the development and persistence of obesity and related co-morbidities into adulthood [8].

Current instruments for the measurement of HRQoL in children and adolescents can be classified into two groups depending on whether health status dimensions are weighted by populations’ preferences. The first and also the most widely used group in practice is non-preference based measures [9], mainly including the Pediatric Quality of Life Inventory™ (PedsQL™) [10], the Child Health Questionnaire™ (CHQ) [11] and the KIDSCREEN [12]. A simple summative scoring system is usually used to generate the HRQoL value for such measures (i.e. each dimension is given an equal weight). The second group is preference-based HRQoL instruments, which have both the ability to ‘measure’ and ‘value’ health status [13]. The outcome of preference-based HRQoL is an index of the strength of preference for a health state, i.e. the health state ‘utility’ which is measured by the utility score, a (0.0–1.0) scale where 1.0 and 0.0 represent full health and dead, respectively. Utilities allow for the comparison of interventions which have differential effects on HRQoL. Such preference based instruments are suitable for application in economic evaluation, specifically within cost utility analysis (CUA) which has been increasingly applied in evaluating public health interventions for children and adolescents [5]. Presently there are four popular preference-based generic instruments available for young people: the Health Utilities Index Mark 2 (HUI2) and Mark 3 (HUI3) [14], the European Quality of Life 5 Dimension Youth version (EQ-5D-Y) [15], [16], the Assessment of Quality of Life 6-Dimension (AQoL-6D) [17], and the Child Health Utility 9D (CHU9D) [18], [19].

The majority of pediatric HRQoL measures were developed and validated in English-speaking countries and there is a paucity of measures currently available in other societies, including China, the most populous country in the world. Currently, the only available HRQoL instrument validated for use with the Chinese children is the non-preference based PedsQL™ [20]–[22]. Considering the population size and rapid transition in China, there is a particular need to develop or validate a preference based instrument for measuring Chinese children’s HRQoL. In addition, it is important to identify potential influencing factors for HRQoL so as to better design effective public health interventions for young people. It is reported in the literature that physical activity, sedentary behaviour and sleep duration are all significantly associated with HRQoL in young people; however, the evidence from mainland China is still limited [23]–[26]. Hong *et al*. studied the relationship between above health behaviours and depression and found that the presence of depression in students was significantly inversely associated with physical activity and long sleep duration and positively associated with study durations (although insignificant) [27]. Mental health is considered as an important component of HRQoL, This study extends the work conducted by Hong *et al*. to investigate the relationship between health behaviours and overall HRQoL.

The CHU9D instrument has previously been demonstrated to be acceptable, practical and valid for application with children and adolescents aged 7–17 years in two countries (UK and Australia) [28]–[30]. Unlike other generic preference based instruments suitable for application with young people which represent adaptations of instruments originally developed for adults (e.g. the EQ-5D-Y and AQoL-6D Adolescent), the CHU9D was developed from its inception with young people [31] with the aim of exploring how health status affects quality of life. The dimensions contained in the CHU9D were identified from in-depth qualitative interviews with young people with a variety of chronic and acute health problems [18]. For these reasons we chose the CHU9D as our preferred instrument.

This study had two main objectives. The first objective was to investigate the construct validity of a Chinese version of CHU9D (CHU9D-CHN) against self-reported health status. It was hypothesized that better self-reported health status would be associated with higher CHU9D utility scores and vice versa. The second objective was to investigate the associations between physical activity, homework and sleep duration with HRQoL among primary and high school students in Mainland China.

## Methods

### Sample

The CHU9D-CHN pilot study was conducted in May 2013 within urban areas of Nanjing City, the capital of Jiangsu Province, Mainland China. Nanjing is composed of six urban and five suburban districts, with approximately eight million regular residents in 2013. The participants of the CHU9D-CHN survey were primary and high school students aged between 9 and 17 (grades 4–12). The students were recruited using a multi-stage sampling method. In the first step, we randomly chose two from a total of six urban districts. Next, within each chosen urban district we randomly selected one school from primary, junior and senior high schools, respectively. In the final step, one class was randomly chosen from each grade within each selected school. This resulted in a total of eighteen classes with two classes from primary, junior and senior high schools (grades 4–12), respectively. All students (N = 839) within those selected classes were eligible participants. Access to the CHU9D-CHN survey data is available on request via the corresponding author.

Written informed consents were obtained from both the schools and parents/students’ guardians. The academic and ethical committee of Nanjing Municipal Center for Disease Control and Prevention reviewed and approved this study in accordance with the internationally agreed ethical principles for medical research involving human subjects.

### Behaviour measurements and socio-demographic characteristics

Self-reported information regarding children and adolescents’ demographics, health status, physical activity, homework and sleep duration was captured through a paper questionnaire in class. Support for completing the questionnaire was made available on request from researchers/teachers. Socio-demographic variables included within the data analysis were age and gender. Information on parental educational attainment was also captured and included in the data analysis. A dummy variable indicating whether or not the mother and/or father received tertiary qualifications was used to capture the highest level of parental education [25]. Participants were asked to rate their own general health on the day of questionnaire completion using a five-level scale: excellent, very good, good, fair or poor [13].

Information on physical activity was collected using a special sub-questionnaire, in which 24 of the most-often engaged in activities were listed. Each participant was asked to record his/her physical activity before sleeping at night day by day for a consecutive period of 7 days. When doing this, each student simply needed to record the frequency and duration for each listed activity item by item and to report zero for the activity if (s)he did not engage in it. Participants were also invited to add any specific activity items if not listed in the questionnaire. Then, each activity was conveyed into a specific metabolic equivalent (MET) value based on the recently updated compendium of physical activities [32]. The physical activity level was calculated for each participant as the sum of MET values of all type of activities. In this analysis, based on the MET value, the physical activity was grouped into high, medium or lower levels if participants were within the top 25%, middle 50% or the low 25% of the MET distribution.

Sedentary behaviour was measured as the average number of hours spent doing homework per day in the past 7 days (hereafter referred to ‘homework duration’). There could be other measures for sedentary behaviour, however in this pilot only homework duration was considered since this is less studied in the literature. Students were also asked to recall on average how many hours they slept per day in the past 7 days (hereafter referred to ‘sleep duration’). In the descriptive analysis, to illustrate the utility distribution more clearly, the homework and sleep duration was divided into two levels according to the median values within each grade level. In the linear regression analysis, homework or sleep duration was included into the model as a continuous variable.

### CHU9D translation

The CHU9D-CHN in simplified Chinese was created by translating from the original English version by All Graduates Interpreting & Translating (http://allgraduates.com.au), a private company-based in Australia. The translators are accredited by the National Accreditation Authority for Translators and Interpreters Ltd (NAATI). A standardised forward and back translation methodology was utilised. The translated version of the CHU9D instrument was then scrutinised by a multidisciplinary team which consisted of health economists and health service researchers in Australia and China to produce a final Chinese version of the CHU9D, the CHU9D-CHN. The CHU9D-CHN was administered as a component of a paper questionnaire and participants were instructed to self-complete the CHU9D-CHN from the perspective of their own health today (i.e. the day when the paper questionnaire was answered).

### CHU9D scoring

The CHU9D consists of 9 dimensions, including worried, sad, pain, tired, annoyed, schoolwork, sleep, daily routine, and ability to join in activities. Within each dimension, there are 5 different levels indicating increasing levels of severity. The instrument was scored using both the UK adult general population standard gamble (SG) scoring algorithm [33] and an Australian adolescent specific best worst scaling (BWS) algorithm [13]. Both algorithms are preference based and generate utility values on the 0.0 to 1.0 quality adjusted life year (QALY) scale, and are therefore suitable for application in the measurement and valuation of health benefits for economic evaluation.

### Analysis

The distribution of utility scores was firstly tested for normality using the Shapiro-Francia test. The Bland-Altman plot was used to show the agreement of utility scores based on SG and BWS scoring methods. The construct validity of CHU9D-CHN was assessed by studying the hypothesized relationships between self-reported health status and utility scores. Specifically, it was expected that participants with a better self-reported health status would have higher utility scores, on average. As utility scores were not normally distributed, two non-parametric tests (Mann Whitney U test and Kruskal Wallis test) were used to compare utility scores between groups. The chi-square test was used to test whether the frequency of responses to CHU9D-CHN instrument between/among sub-groups were independent [34]. Linear regression analysis was conducted to study the potential associations between selected behaviours (predictor variables) and the utility score (dependent variable) after controlling for socio-demographic variables (age, gender and parental education). Considering the two-level nature of the survey data that students (level 1) are nested within schools (level 2), a two-level random-intercept model was used [35]. Except for the Bland-Altman plot which was conducted using MedCalc version 12.7.2 (MedCalc Software bvba, Ostend, Belgium), all other analyses were performed in Stata version 12.1 (StataCorp LP, College Station, Texas, USA).

## Results

The characteristics of participants are presented in Table 1. Of the 839 eligible participants, 815 completed the questionnaire (participating rate 97.1%), with 31.8% in grades 4–6 (primary school students), 37.1% grades 7–9 (junior high school students) and 31.1% grades 10–12 (senior high school student). The mean (range) age was 14.1 (9–19) years. A higher proportion of participants were boys (54.5%) and coming from a family with at least one parent had a college diploma (53.1%). Participants did physical activity at the level of 1932.1 (standard deviation, SD = 1811.6) METs, spent 2.3 hours (SD = 1.2) on homework and slept 7.6 (SD = 1.2) hours each day on average. Overall, 90.3% of participants self-reported health status was good, very good or excellent.

**Table 1:** Characteristics of participants.

Characteristic	All (N = 815)
Age, years	
mean (SD)	14.1 (2.5)
Gender	
Boys, n (%)	444 (54.5)
Girls, n (%)	371 (45.5)
Grade level	
4 (∼10 years old), *n* (%)	81 (9.9)
5 (∼11 years old), *n* (%)	90 (11.0)
6 (∼12 years old), *n* (%)	89 (10.9)
7 (∼13 years old), *n* (%)	104 (12.8)
8 (∼14 years old), *n* (%)	98 (12.0)
9 (∼15 years old), *n* (%)	100 (12.3)
10 (∼16 years old), *n* (%)	93 (11.4)
11 (∼17 years old), *n* (%)	100 (12.3)
12 (∼18 years old), *n* (%)	60 (7.4)
Parental education	
Mother and/or father has tertiary qualification, n (%)	433 (53.1)
Child Health Utility 9D utility scores, SG	
mean (SD)	0.81 (0.11)
Child Health Utility 9D utility scores, BWS	
mean (SD)	0.75 (0.14)
Total metabolic equivalent (MET) level	
mean (SD)	1932.1 (1811.6)
Average homework hours per day in the last 7 days	
mean (SD)	2.3 (1.2)
Average sleep duration hours per day in the last 7 days	
mean (SD)	7.6 (1.2)
Self-assessed health status	
Excellent, n (%)	276 (33.9)
Very good, n (%)	315 (38.7)
Good, n (%)	145 (17.8)
Fair or poor, n (%)	79 (9.7)

SD - standard deviation; SG - standard gambling method; BWS - best worst scaling method.

**Figure 1:**
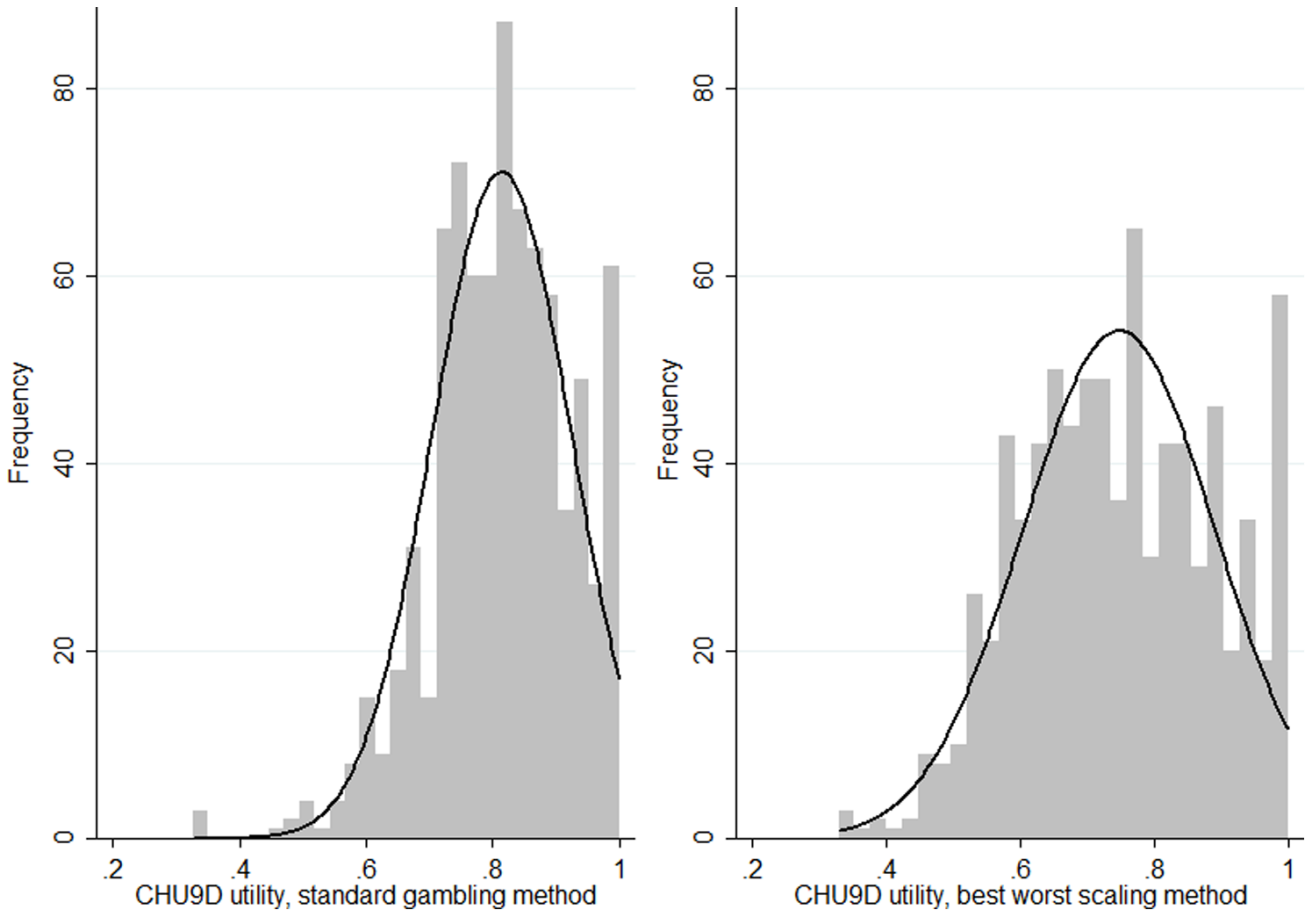
Distributions of Child Health Utility 9D (CHU9D) utilities.

**Figure 2:**
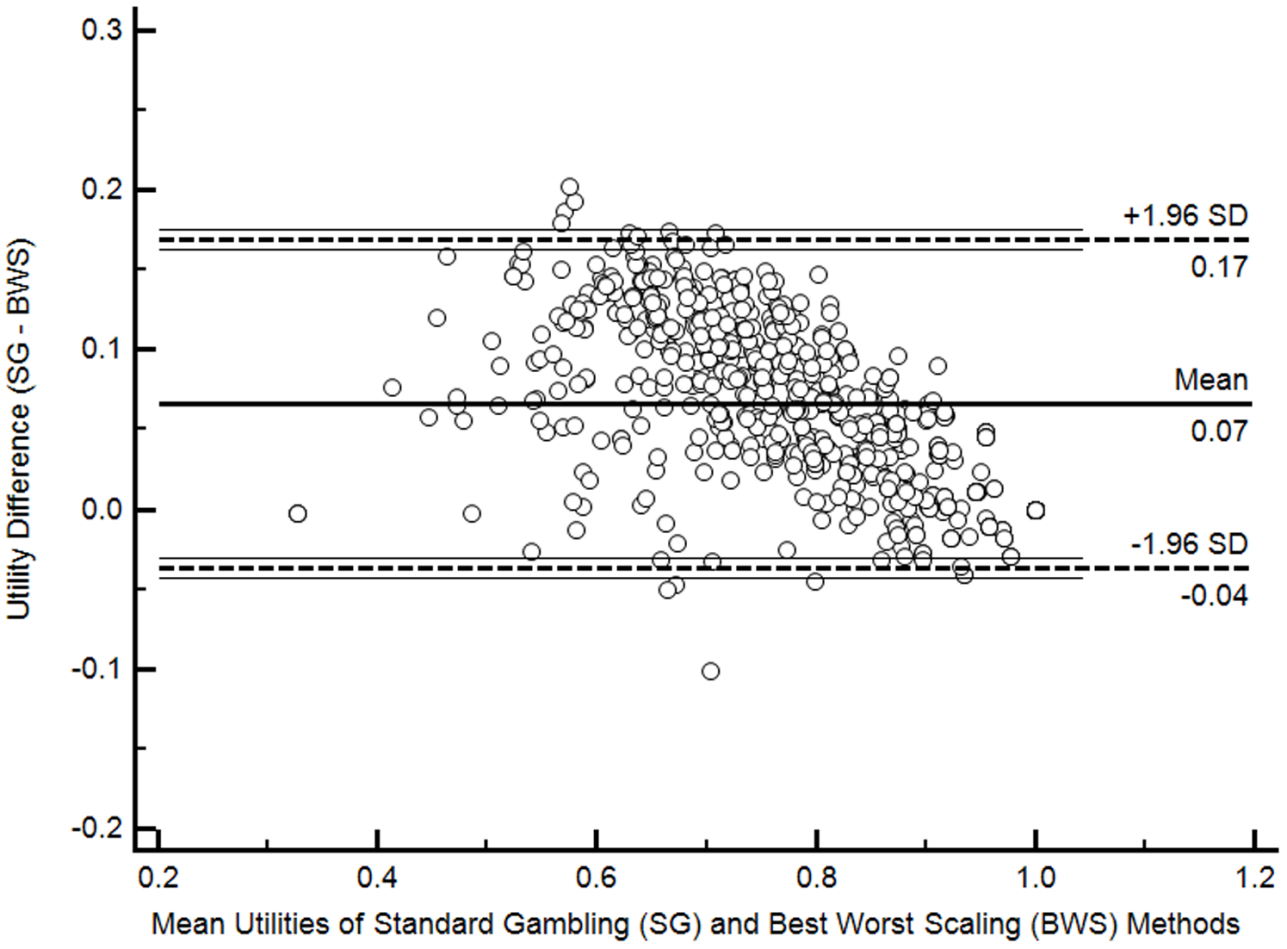
Bland-Altman plot of differences in Child Health Utility 9D (CHU9D) utilities from two scoring methods.

The mean (range) CHU9D utility scores were 0.81 (0.33–1.0) and 0.75 (0.33–1.0) based on the SG and BWS scoring algorithms, respectively. Approximately 6.4% of participants were classified in full health (i.e. utility = 1) based on the both scoring algorithm. The distribution of utilities based on two scoring algorithms is presented in Figure 1. The CHU9D utility scores were not normally distributed regardless of which scoring algorithm was adopted (Shapiro-Francia test statistics, p<0.001). The Bland-Altman plot shown in Figure 2 suggests that the utility scores generated from application of the two scoring algorithms had a high level of agreement (95% limits of agreement (LOA), −0.04 to 0.17; 1.72% of observations outside the LOA). In addition, the SG method tends to produce higher utilities than the BWS method (mean difference 0.07, 95% confidence interval 0.06 to 0.07, P<0.001, Figure 2).

Mean CHU9D utilities by self-reported health status, physical activity, homework hours, and sleep duration are presented in Table 2. Since the sample size of participants with a fair or poor self-reported health was small (n = 66 and n = 13, respectively), we grouped these two categories together in the analysis. CHU9D utilities discriminated well according to self-reported health status and better health status was associated with higher utility no matter which scoring methods were used (both P<0.001): mean utilities ranged from 0.88 for those who were in excellent health to 0.70 in fair or poor health when the SG scoring algorithm as adopted, whilst corresponding mean utilities based on the BWS scoring method ranged from 0.83 to 0.61. Statistically significantly positive correlations were observed between a higher level of physical activity in the previous week, shorter homework hours, longer sleeping hours and a higher mean utility at the 1‰ level.

**Table 2:** Mean utility scores of Child Health Utility 9D by self-reported health and behaviour domains.

	N	Standard Gamble method			Best Worst Scaling method		
		Mean	SD	*P* value†	Mean	SD	*P* value†
Self-reported health				<0.001			<0.001
Excellent	276	0.88	0.10		0.83	0.13	
Very good	315	0.81	0.09		0.74	0.12	
Good	145	0.76	0.10		0.67	0.12	
Fair or poor	79	0.70	0.11		0.61	0.12	
Physical activity level				<0.001			<0.001
Low (MET ≤725)	198	0.79	0.12		0.72	0.15	
Medium (725< MET ≤2575)	413	0.81	0.10		0.74	0.13	
High (MET >2575)	204	0.84	0.11		0.79	0.14	
Homework hours in the last 7 days				<0.001			<0.001
≤ median time‡	492	0.83	0.11		0.76	0.14	
> median time‡	323	0.79	0.11		0.72	0.14	
Average sleep duration hours in the last 7 days				<0.001			<0.001
≤ median time‡	536	0.80	0.11		0.73	0.14	
> median time‡	279	0.85	0.10		0.79	0.14	

MET: metabolic equivalent.

† Mann Whitney U test for 2 groups; Kruskal Wallis test for more than 2 groups.

‡ Median values were calculated within each grade level.

The frequency of responses to the CHU9D-CHN dimension levels are presented in Table 3. Amongst the 9 CHU9D-CHN dimensions, the majority of students reported themselves at the best level of daily routine (86.3%), followed by pain (65.5%), sad (58.8%). Less than half of students reported themselves at the best level of ability to join in activities (47.9%), worried (40.9%), schoolwork/homework (38.9%), sleep (38.9%), annoyed (33.1%), and tired (19.3%). Across the three levels of physical activity, there were statistically significant difference on 6 dimensions, including ability to join in activities (P<0.001), tired (P<0.001), worried (P = 0.02), annoyed (P = 0.02), sleep (P = 0.03), and daily routine (P = 0.04). Six dimensions were found to have statistically different distributions (P<0.05) for students within two groups of homework duration; those are schoolwork/homework (P = 0.001), sad (P = 0.006), worried (P = 0.01), sleep (P = 0.03), activity (P = 0.03), and tired (P = 0.04). For sleep duration, the difference was statistically significant for all 9 dimensions (P = 0.04 for ability to join in activities, all else P≤0.01). It can be seen from Table 3 that students who were more physical active, spent less hours on homework, or maintained longer sleep durations were more likely to classify themselves at better dimension levels.

**Table 3:** Responses to the Child Health Utility 9D (CHU9D), frequency (%).

CHU9D dimensions and levels	ALL	Physical activity			Homework		Sleep	
		Low	Medium	High	≤ median time†	> median time†	≤ median time†	> median time†
		N = 198	N = 413	N = 204	N = 492	N = 323	N = 536	N = 279
**1. Worried**		P = 0.02			P = 0.01		P = 0.001	
I don’t feel worried today	40.9	37.4	38.3	49.5	45.1	34.4	38.3	45.9
I feel a little bit worried today	34.9	32.3	37.1	32.8	33.9	36.2	33.0	38.4
I feel a bit worried today	15.0	20.7	14.5	10.3	13.4	17.3	17.0	11.1
I feel quite worried today	6.0	4.6	7.3	4.9	5.1	7.4	7.3	3.6
I feel very worried today	3.3	5.1	2.9	2.5	2.4	4.6	4.5	1.1
**2. Sad**		P = 0.19			P = 0.006		P = 0.001	
I don’t feel sad today	58.8	55.6	56.7	66.2	63.6	51.4	54.9	66.3
I feel a little bit sad today	25.5	25.8	28.8	18.6	23.8	28.2	25.9	24.7
I feel a bit sad today	9.1	11.1	8.0	9.3	7.5	11.5	10.5	6.5
I feel quite sad today	2.9	3.0	2.7	3.4	2.0	4.3	3.9	1.1
I feel very sad today	3.7	4.6	3.9	2.5	3.1	4.6	4.9	1.4
**3. Pain**		P = 0.10			P = 0.10		P = 0.002	
I don’t have any pain today	65.5	58.6	66.6	70.1	68.9	60.4	61.8	72.8
I have a little bit of pain today	21.5	23.7	21.6	19.1	20.3	23.2	22.2	20.1
I have a bit of pain today	7.2	11.1	6.8	4.4	5.9	9.3	8.4	5.0
I have quite a lot of pain today	3.3	2.5	3.2	4.4	2.9	4.0	4.7	0.7
I have a lot of pain today	2.5	4.0	1.9	2.0	2.0	3.1	3.0	1.4
**4. Tired**		P<0.001			P = 0.04		P<0.001	
I don’t feel tired today	19.3	15.7	15.5	30.4	21.1	16.4	14.4	28.7
I feel a little bit tired today	31.3	27.3	34.1	29.4	32.7	29.1	30.6	32.6
I feel a bit tired today	23.1	26.3	24.5	17.2	23.4	22.6	23.5	22.2
I feel quite tired today	15.5	15.2	16.5	13.7	14.0	17.7	18.8	9.0
I feel very tired today	10.9	15.7	9.4	9.3	8.7	14.2	12.7	7.5
**5. Annoyed**		P = 0.02			P = 0.12		P<0.001	
I don’t feel annoyed today	33.1	30.8	29.3	43.1	34.8	30.7	30.0	39.1
I feel a little bit annoyed today	39.8	36.9	42.4	37.3	41.5	37.2	37.7	43.7
I feel a bit annoyed today	16.2	17.7	17.9	11.3	14.6	18.6	19.0	10.8
I feel quite annoyed today	5.4	8.1	5.1	3.4	4.5	6.8	6.3	3.6
I feel very annoyed today	5.5	6.6	5.3	4.9	4.7	6.8	6.9	2.9
**6. Schoolwork/homework**		P = 0.61			P = 0.001		P = 0.001	
I have no problems with my schoolwork/homework today	38.9	35.9	38.7	42.2	43.3	32.2	34.5	47.3
I have a few problems with my schoolwork/homework today	44.5	45.0	43.6	46.1	43.5	46.1	46.6	40.5
I have some problems with my schoolwork/homework today	12.5	14.1	13.6	8.8	11.0	14.9	13.4	10.8
I have many problems with my schoolwork/homework today	2.6	3.0	2.4	2.5	1.6	4.0	3.5	0.7
I can’t do my schoolwork/homework today	1.5	2.0	1.7	0.5	0.6	2.8	1.9	0.7
**7. Sleep**		P = 0.03			P = 0.03		P<0.001	
Last night, I had no problems sleeping	38.9	30.3	38.3	48.5	40.5	36.5	31.0	54.1
Last night, I had a few problems sleeping	31.5	33.3	32.5	27.9	33.9	27.9	31.7	31.2
Last night, I had some problems sleeping	24.5	31.3	24.5	18.1	21.3	29.4	30.0	14.0
Last night, I had many problems sleeping	2.7	3.0	2.4	2.9	2.6	2.8	3.9	0.4
Last night, I couldn’t sleep at all	2.3	2.0	2.4	2.5	1.6	3.4	3.4	0.4
**8. Daily routine**		P = 0.04			P = 0.12		P = 0.01	
I have no problems with my daily routine today	86.3	84.9	84.5	91.2	87.4	84.5	83.6	91.4
I have a few problems with my daily routine today	10.7	9.6	12.4	8.3	10.2	11.5	13.1	6.1
I have some problems with my daily routine today	1.6	3.5	1.5	0.0	1.8	1.2	1.7	1.4
I have many problems with my daily routine today	0.9	0.5	1.2	0.5	0.4	1.6	0.8	1.1
I can’t do my daily routine today	0.6	1.5	0.5	0.0	0.2	1.2	0.9	0.0
**9. Able to join in activities**		P<0.001			P = 0.03		P = 0.04	
I can join in with any activities today	47.9	37.9	46.5	60.3	51.4	42.4	44.8	53.8
I can join in with most activities today	21.0	19.7	21.3	21.6	20.1	22.3	20.7	21.5
I can join in with some activities today	13.3	9.6	17.0	9.3	12.6	14.2	15.5	9.0
I can join in with a few activities today	10.7	19.2	9.2	5.4	10.6	10.8	11.4	9.3
I can join in with no activities today	7.2	13.6	6.1	3.4	5.3	10.2	7.7	6.5

*P* value reported in the table based on chi-square test.

† Median values were calculated within each grade level.

The relationships between physical activity, homework hours, sleep duration and utilities are reported in Table 4. After adjustment for age, gender and parental education (hereafter ‘adjusted mean utilities’), compared to those grouped as the lowest level for physical activity, those with high level of physical activity were found to have a significant higher mean utility (0.023 or 0.029 depending on whether SG or BWS scoring algorithms was adopted, both P<0.05), while the medium level physically active students had slightly higher utilities, although the difference was insignificant. Each additional hour of doing homework was associated with a decrease in mean utility of 0.019 or 0.021 based on the SG or BWS scoring algorithms respectively (both P<0.01). On average, one additional hour of sleep was significantly associated with an increase in mean utility of 0.032 or 0.040 using SG or BWS scoring algorithms respectively (both P<0.01).

**Table 4:** Adjusted mean differences in Child Health Utility 9D (CHU9D) utility scores.

	Dependent Variable: CHU9D utility scores, standard gambling method			Dependent Variable: CHU9D utility scores, best worst scaling method		
	(1)	(2)	(3)	(4)	(5)	(6)
Physical activity level: medium	0.009			0.010		
	[0.009]			[0.012]		
Physical activity level: high	0.023**			0.029**		
	[0.011]			[0.014]		
Homework hours in the last 7 days		−0.019***			−0.021***	
		[0.004]			[0.005]	
Sleep duration hours in the last 7 days			0.032***			0.040***
			[0.004]			[0.005]
Age	−0.007***	−0.005**	−0.001	−0.012***	−0.009***	−0.004
	[0.003]	[0.003]	[0.003]	[0.003]	[0.003]	[0.003]
Gender: Male	0.014*	0.014*	0.008	0.022**	0.023**	0.016*
	[0.007]	[0.007]	[0.007]	[0.009]	[0.009]	[0.009]
Parental education	−0.007	−0.006	−0.006	−0.000	0.000	0.001
	[0.009]	[0.009]	[0.008]	[0.011]	[0.011]	[0.011]
No. of Students	815	815	815	815	815	815
No. of Schools	5	5	5	5	5	5

Standard errors in brackets.

***p<0.01,

**p<0.05,

*p<0.1. The reference level for physical activity is the low level.

## Discussion

This study assessed the construct validity of the Chinese version of the CHU9D against self-reported health status in a child and adolescent population in China. The CHU9D-CHN was developed through translation by an accredited professional interpreting and translating company based in Australia by government accredited translators. Since a Chinese-population specific scoring algorithm is not yet available, the original UK SG scoring algorithm and a more recently developed Australian BWS algorithm were adopted to score the instrument. Application of the CHU9D-CHN indicated that physical activity, homework and sleep duration were all significantly associated with HRQoL for children and adolescents in Mainland China.

There was no evident ceiling effect for the CHU9D-CHN instrument based on the current BWS and SG algorithms, nor any evident ceiling effects for the majority of CHU9D-CHN dimensions. The most noteworthy ceiling effect was observed on the daily routine dimension (86.3%), which indicates the potential for reduced discriminatory power for this dimension in this student sample. Construct validity was assessed by comparing utility scores with self-reported health status. Although the mean utility generated through utilisation of the SG algorithm were slightly higher than the ones generated through the BWS algorithm, the results suggested that CHU9D-CHN discriminated well in relation to participants’ self-reported health status and that better health status was significantly associated with a higher utility score no matter which scoring method was adopted.

The significantly positive association between physical activity and HRQoL observed in this study is consistent with what has been recently reported using survey data for children and adolescents in Australia [24]–[26]. So far the evidence on the relationship between homework duration and HRQoL is very limited. To our knowledge, this is the first study internationally to report utility scores associated with homework duration for children and adolescents. The mean utility loss associated with an additional homework hour was 0.02. Opposite to what has been reported in this study, Gopinath et al. [25] found that among four sedentary behaviours (television viewing, video-game usage, computer usage and homework), students that spent more time doing homework had higher HRQoL. For the Australian study, the mean homework hours were 1.5 hours whilst the mean homework hours were 2.3 hours in our study. The opposite results may be owing to the potential non-linear relationship between homework duration and HRQoL. In addition, the different cultural and lifestyle differences in the Australian vs. Chinese contexts could also be an explanation for this finding. More empirical evidence is required before we can draw stronger conclusions about the relationship between homework duration and HRQoL.

The association between sleep duration and HRQoL has gained increasingly attention in recent years. Chiu et al. [36] studied a young resident sample (aged 16–34) in rural China (Mianyang City, Sichuan Province). Using the World Health Organization (WHO) Quality of Life Schedule-Brief [37], the authors found that being a short sleeper (<7 hours/day) was associated with lower HRQoL in the physical psychological and environmental domains, whilst being a longer sleeper were associated with higher HRQoL in the environmental domain. Our study found consistent results that longer sleeping hours (>8 hours/day) were associated with higher HRQoL in general for both children and adolescents. The magnitude of mean utility associated with an additional hour of sleep in this study was larger than that reported for a recent study based on Australian children and adolescent sample (0.04 vs. 0.01, both based on CHU9D instruments and scored using BWS method) [26]. The positive relationship between sleep duration and HRQoL has also been reported for older Chinese adults in Hong Kong [38]. The authors found that long sleepers (≥5.5 hours/night) had significantly better HRQoL than short sleepers in all eight SF-36® domains (with the largest/smallest difference in body pain/role-emotional domain).

A particular strength of this study is that the students were randomly recruited using a multi-stage sampling approach. Further considering the high participating rate (97.1%), the potential impact of self-selection bias would be minimised. The study also has a number of limitations which would benefit from further research. Firstly, whilst the translation was undertaken by a professional translation agency for use as a pilot instrument in this study, a fully validated translation and linguistic validation process is the ideal and industry standard. This consists of several stages, including forward and back translations, reconciliations, cognitive debriefing and developer review [39]. As this study has shown promise with the pilot translation, the research team is now actively engaged in planning the development of a fully validated version of the instrument. Secondly, the scoring algorithms applied for CHU9D-CHN are derived from UK and Australian general population samples. To better reflect the Chinese population preference for health status, it is important to derive a scoring algorithm for the CHU9D-CHN from the Chinese population. Thirdly, more evidence is needed to validate the CHU9D-CHN in both community based general population samples and patient samples in Mainland China. It would be valuable to administrate the CHU9D-CHN simultaneously with another validated pediatric HRQoL measure (such as PedsQL™) to further study the construct validity. Fourthly, it would be helpful to conduct further research to determine the minimum clinically importance difference (MCID) for CHU9D-CHN utility scores to help determine whether the statistically significant difference observed are also likely to be clinically significant. In the literature, a difference of 0.03 on the 0–1 death full health QALY scale is considered to be important for other preference based instruments used for adults [40]. It is unclear whether that criterion could be directly applied in assessing the differences in utility scores in this new instrument for young people. Finally, we cannot draw any causal conclusion between physical activity, homework and sleep durations and HRQoL based on this cross-sectional survey data. HRQoL may also impact on individual’s behaviours. In addition, there could be other unobservable variables influencing both HRQoL and behaviour indicators, such as personality traits. Without careful handling of the above potential reverse causality and omitted variable biases, the conclusion of this study should be explained as associations only.

## Conclusion

The findings from this pilot study demonstrate the construct validity of a Chinese translation of the CHU9D for measuring and valuing the HRQoL of children and adolescents in China. Levels of self-reported physical activity, homework and sleep time were important influencers of utility scores. Future research should undertake a full translation and linguistic validation of the CHU9D into Chinese to further explore the validity of the measure in both community-based general population samples and patient samples in Mainland China and the development of a Chinese population specific scoring algorithm.
